# Future risk of metabolic syndrome in women with a previous LGA delivery stratified by gestational glucose tolerance: a prospective cohort study

**DOI:** 10.1186/s12884-018-1958-z

**Published:** 2018-08-10

**Authors:** Heidi Hakkarainen, Hanna Huopio, Henna Cederberg, Raimo Voutilainen, Seppo Heinonen

**Affiliations:** 1 Department of Obstetrics and Gynecology, Kuopio University Hospital, Puijonlaaksontie 2, P.O.B 100, 70029 KYS, Kuopio, Finland; 2 Institute of Clinical Medicine, School of Medicine, University of Eastern Finland, P.O.B 1627, 70211 Kuopio, Finland; 3 Department of Pediatrics, Kuopio University Hospital, P.O.B 100, 70029 KYS, Kuopio, Finland; 4 Department of Medicine, Helsinki University Hospital, Jorvi Hospital, P.O.B 800, 00029 HUS, Helsinki, Finland; 5 Faculty of Medicine, Center for Life Course Epidemiology and Systems Medicine, University of Oulu, P.O.B 8000, 90014 Oulu, Finland; 6 Department of Pediatrics, University of Eastern Finland, P.O.B 1627, 70211 Kuopio, Finland; 7 Department of Obstetrics and Gynecology, Helsinki University Central Hospital, P.O.B 140, 00029 HUS, Helsinki, Finland; 8 Department of Obstetrics and Gynecology, University of Helsinki, P.O.B 3, 00014 Helsinki, Finland

**Keywords:** Gestational diabetes mellitus, Metabolic syndrome, Large-for-gestational-age, Birth weight

## Abstract

**Background:**

Whether the delivery of a large-for-gestational-age (LGA) infant predicts future maternal metabolic syndrome (MetS) is not known. To this aim, we investigated the incidence of MetS and its components in women with or without a history of gestational diabetes mellitus (GDM) with a view to the birth weight of the offspring.

**Methods:**

Eight hundred seventy six women treated for their pregnancies in Kuopio University Hospital in 1989–2009 underwent a follow-up study (mean follow-up time 7.3 (SD 5.1) years), of whom 489 women with GDM and 385 normoglycemic controls. The women were stratified into two groups according to the newborn’s birth weight: 10-90th percentile (appropriate-for-gestational-age; AGA) (*n* = 662) and > 90th percentile (LGA) (*n* = 116). MetS and its components were evaluated in the follow-up study according to the International Diabetes Federation criteria.

**Results:**

LGA vs. AGA delivery was associated with a higher incidence of MetS at follow-up in women with a background of GDM (54.4% vs. 43.6%), but not in women without GDM.

**Conclusion:**

An LGA delivery in women with GDM is associated with a higher risk of future MetS and this group is optimal to study preventive measures for MetS. In contrast, an LGA delivery after a normoglycemic pregnancy was not associated with an increased future maternal MetS risk.

## Background

Gestational diabetes mellitus (GDM) increases the risk of obstetric complications, largely due to fetal overgrowth. In addition, GDM is associated with an increased risk of developing type 2 diabetes (T2DM) [1–3], metabolic syndrome (MetS) and cardiovascular diseases (CVD) [4–8] after the pregnancy. The key pathophysiological defects underlying the increased cardiometabolic morbidity after GDM pregnancy include chronic insulin resistance and impaired insulin secretion, together with visceral obesity, hypertension and dyslipidemia [9]. Disturbance in glucose metabolism is considered to be a major cause for a large-for-gestational-age (LGA) delivery [10], albeit many environmental and genetic factors are also likely to play a role. In particular, maternal pre-pregnancy body mass index (BMI) and gestational weight gain have been shown to be independent determinants of the infant birth weight [11–13]. Women with pre-pregnancy overweight and obesity were at 1.5-fold and 2-fold increased risk of delivery of an LGA infant, respectively [12]. Furthermore, maternal metabolic factors including decreased high-density lipoprotein (HDL) cholesterol, increased triglycerides [14] and insulin have previously been shown to be independent determinants of fetal macrosomia [15]. In continuum, infant born LGA and exposed to an intrauterine environment of diabetes or maternal obesity have also been shown to be at an increased risk of developing MetS later in their lives [16].

We therefore hypothesized that a previous LGA delivery would be associated with an increased risk of incident MetS in the mother after the pregnancy. To this aim, we investigated the incidence of MetS and its components in women with and without GDM by groups of different birth size.

## Methods

This hospital register-based cohort study included women whose pregnancies were treated in Kuopio University Hospital, Finland, in 1989–2009. Women who had the diagnosis of GDM and a random sample of normoglycemic women, both groups with completed oral glucose tolerance test (OGTT) during pregnancy, were contacted by a letter and invited for the study. A total of 489 women with GDM and 385 women with normal OGTT result during pregnancy attended the follow-up study. 1234 women did not reply or declined to participate in the study.

The women with and without GDM were classified based on the birth weight of the newborn: between 10-90th percentile (appropriate-for-gestational-age; AGA) (*n* = 662) and over 90th percentile (LGA) (*n* = 116). The women without GDM and delivery of an AGA infant served as a control group. In this study, LGA was defined as sex-specific birth weight for gestational age above the 90th percentile on the current Finnish newborn growth charts [17].

### Data collection during pregnancy

In Finland, cost-free maternity care is offered to all pregnant women. The women considered to be at risk of GDM underwent 2-h OGTT (75 g glucose after overnight fasting) between the 24th and 28th weeks of gestation if one or more following factors were present: age over 40 years, BMI ≥ 25 kg/m^2^, prior GDM or a history of a macrosomic delivery, glucosuria, suspected fetal macrosomia in the current pregnancy. The diagnostic criteria of GDM were as follows: until September 2001 the lower limits of abnormal fasting, 1-h and 2-h capillary whole-blood glucose 4.8, 10.0 and 8.7 mmol/l and since September 2001 the lower limits of fasting, 1-h and 2-h capillary plasma glucose 4.8, 11.2 and 9.9 mmol/l as per contemporary guidelines. For the women with more than one delivery during the study period, the first pregnancy with an abnormal OGTT result was selected as the index pregnancy. The women with GDM were seen regularly in the Prenatal Outpatient Clinic in Kuopio University Hospital and they received dietary advice, regular blood glucose monitoring and insulin treatment when necessary. The hospital register included data on maternal characteristics and pregnancy risk factors, complications, pregnancy outcome, and on the neonatal period of the offspring. The women with overt T2DM at the time of pregnancy or type 1 diabetes mellitus (T1DM) diagnosed after the index pregnancy, and those with a multiple pregnancy were excluded to eliminate confounding factors.

### The follow-up study

The participants were recruited to the follow-up study between 2006 and 2009. The women underwent laboratory tests, body composition and blood pressure measurements, and answered questionnaires concerning their family history and health behavior. All participants underwent a 2-h OGTT (75 g of glucose). MetS was diagnosed by waist circumference ≥ 80 cm, and at least two of the following four criteria in accordance with the International Diabetes Federation (IDF) 2005 criteria [18]: blood pressure ≥ 130/85 mmHg, fasting plasma glucose ≥5.6 mmol/l, serum triglycerides ≥1.7 mmol/l, and HDL cholesterol ≤1.29 mmol/l. These criteria were selected since they are similar to the current care guidelines of MetS in Finland. The women using medication for hyperglycemia, hypertension or dyslipidemia were included in the analysis for the components of MetS.

Height was measured to the nearest 0.5 cm and weight to the nearest 0.1 kg. Body mass index (BMI) was calculated as weight (kg) divided by the height (m) squared. Waist circumference (at the midpoint between the lateral iliac crest and the lowest rib) was measured to the nearest 0.5 cm.

### Laboratory determinations

Plasma glucose was measured by an enzymatic hexokinase photometric assay (Konelab Systems reagents; Thermo Fischer Scientific, Vantaa, Finland). LDL-cholesterol, HDL-cholesterol and total triglycerides were measured by enzymatic colorimetric tests (Konelab Systems reagents).

### Statistical analyses

The statistical analyses were conducted using SPSS version 23 (SPSS Inc., Chicago, IL). *P* < 0.05 was considered statistically significant. The results were given as the mean ± SD or number of cases and percentages. Statistical differences in categorical variables between the study and comparison groups were evaluated using the χ^2^ test. Anthropometric and biochemical continuous variables were analyzed using Student’s t-test, and log-transformed variables were used to correct for their skewed distribution when appropriate. Since the diagnosis of GDM was based on slightly different criteria depending on the origin of the blood during the data collection, a correlation coefficient was used to convert all values to correspond venous plasma levels. The correlation coefficient was based on the information from the Department of Clinical Chemistry at Kuopio University Hospital.

This study was approved by the local Ethics Committee of the Kuopio University Hospital in accordance with the Helsinki Declaration.

## Results

The clinical characteristics of the study groups stratified according to the birth weight of the offspring in index pregnancy and at the follow-up are shown in Table 1. Women with GDM were older in both birth weight categories as compared to controls during the index pregnancy. Women in both GDM groups and the women without GDM but with an LGA delivery were of higher weight and more frequently multiparous than the controls. Women with LGA infants had more frequently a history of prior child’s birth weight over 4000 g as compared to than those with AGA offspring. Furthermore, women with GDM and an LGA delivery had more often a prior spontaneous abortion. The study groups did not differ in the rate of prior cesarean section. The incidence of pre-eclampsia was higher in women with GDM. No significant differences were observed in gestational age at birth between the study groups.

**Table 1:** Clinical characteristics of the controls and GDM subjects in index pregnancy and at the follow-up study stratified according to the offspring’s birth weight

Offspring’s birth weight	AGA (10-90th percentile)		LGA (>90th percentile)	
	Mean ± SD or %		Mean ± SD or %	
	No GDM	GDM	No GDM	GDM
	(Controls)	(Group 1)	(Group 2)	(Group 3)
Number of subjects	286	376	48	68
At the index pregnancy				
Age (yrs)	29.5 ± 5.4	31.8 ± 6.0**	30.6 ± 5.0	32.6 ± 6.3**
Primiparity (%)	53.0	35.0**	34.1*	22.2**
Pre-pregnancy BMI (kg/m^2^)	23.8 ± 3.8	26.4 ± 5.0**	25.7 ± 3.5*	26.7 ± 4.1**
Family history of diabetes (%)	69.4	81.4**	75.0	80.9
Prior child’s birth weight > 4000 g (%)	25.4	25.6	43.8*	60.4**
Prior spontaneous abortion (%)	16.8	19.9	18.8	35.3*
Prior cesarean section (%)	5.9	9.6	16.7	7.4
Gestational age (d)	280 ± 11	279 ± 9	279 ± 11	278 ± 8
Pre-eclampsia (%)	1.4	5.3*	2.1	5.9*
Birth weight (g)	3595 ± 385	3596 ± 406	4365 ± 424**	4421 ± 370**
Placental-fetal mass ratio (%)	17.1 ± 3.0	17.5 ± 3.0	20.6 ± 15.8**	17.9 ± 2.5*
Low Apgar score 1 min < 7 (%)	1.7	5.9*	6.3	4.4
At the follow-up study				
Follow-up time (yrs)	8.5 ± 5.5	5.3 ± 4.3**	7.4 ± 5.4	6.2 ± 4.9*
Age at follow-up (yrs)	38.4 ± 6.4	37.4 ± 7.2	38.3 ± 5.8	39.1 ± 7.5
BMI (kg/m^2^)	26.5 ± 4.9	28.3 ± 5.7**	27.9 ± 4.8*	29.2 ± 4.9**
Weight gain during the follow-up time (kg)	5.7 ± 7.6	3.6 ± 7.9	4.3 ± 7.6	5.6 ± 9.3

*GDM* gestational diabetes mellitus, *BMI* body mass index, *AGA* appropriate for gestational age, *LGA* large for gestational age

All groups compared to controls separately

^*^p < 0.05

^**^p < .0001

At the time of the follow-up study, the women with GDM in both birth weight categories had shorter follow-up time. However, no difference in the mean age of the women was observed between the study groups. The women with GDM and the ones without GDM but with an LGA delivery were of higher weight than the controls, although the study groups did not differ in weight gain during the follow-up time (Table 1).

The comparison of cardiovascular and metabolic parameters of the study groups at follow-up is shown in Table 2. The women with GDM in both birth weight groups and the women without GDM with an LGA delivery had significantly higher waist circumference than the control group; approximately 80% of the women in those three groups reached the 80 cm waist circumference limit. Both GDM groups had significantly lower HDL levels and higher fasting plasma glucose than the control group, with approximately 50% of the women with GDM exceeding the limit 5.6 mmol/l at the follow-up visit. The mean triglyceride levels were higher in women with GDM in both birth weight categories. However, the study groups did not differ significantly concerning the triglyceride level over 1.7 mmol/l required for MetS criterion. No significant differences were observed in total blood pressure between the study groups, even though the mean systolic blood pressure in both GDM groups and diastolic pressure in the women with GDM and LGA infants was significantly higher as compared to controls (Table 2).

**Table 2:** The components of the metabolic syndrome (MetS) in the study subjects at the follow-up study stratified according to the offspring’s birth weight

Offspring’s birth weight	AGA (10-90th percentile)		LGA (>90th percentile)	
	Mean ± SD or %		Mean ± SD or %	
	No GDM	GDM	No GDM	GDM
	(Controls)	(Group 1)	(Group 2)	(Group 3)
Number of subjects	286	376	48	68
Waist circumference (cm)	85.3 ± 11.6	91.0 ± 13.8**	88.8 ± 10.8*	94.2 ± 12.9**
Waist circumference ≥ 80 cm (%)	64.0	77.6**	81.3*	89.7**
Fasting glucose (mmol/l)	5.3 ± 0.4	5.6 ± 0.8**	5.4 ± 0.4	5.8 ± 0.8**
Fasting glucose ≥5.6 mmol/l (%)	25.2	46.8**	29.2	58.8**
Triglycerides (mmol/l)	1.0 ± 0.6	1.1 ± 0.6*	0.9 ± 0.4	1.2 ± 0.5*
Triglycerides ≥1,70 mmol/l (%)	10.6	15.2	4.2	16.2
HDL cholestrol (mmol/l)	1.5 ± 0.4	1.4 ± 0.4**	1.6 ± 0.3	1.3 ± 0.3**
HDL cholestrol < 1.29 mmol/l (%)	28.3	44.1**	20.8	57.4**
Systolic pressure (mmHg)	122.5 ± 14.3	125.8 ± 14.1*	121.4 ± 10.1	128.4 ± 14.2*
Diastolic pressure (mmHg)	78.3 ± 9.6	79.3 ± 9.2	77.5 ± 7.5	81.3 ± 10.0*
Blood pressure ≥ 130/≥85 mmHg (%)	34.3	38.8	27.1	45.6
Metabolic syndrome (IDF) (%)	24.5	43.6**	18.8	54.4**

*GDM* gestational diabetes mellitus, *BMI* body mass index, *AGA* appropriate for gestational age, *LGA* large for gestational age

All groups compared to controls separately

*p < 0.05

**p < .0001

**Fig. 1:**
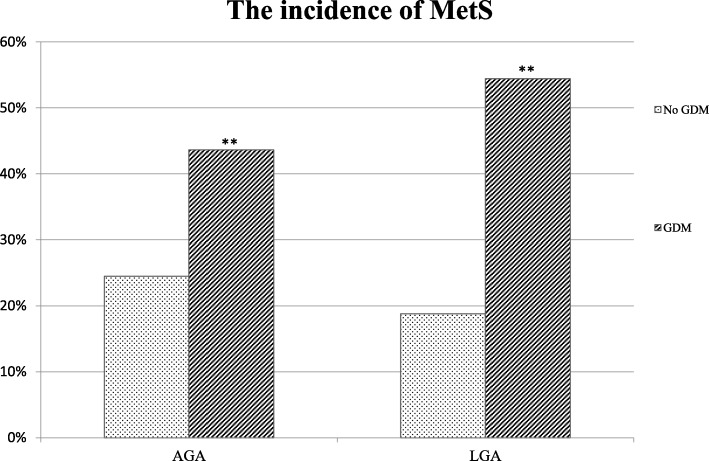
The incidence of the metabolic syndrome among the study groups at the follow-up visit according to the birth weight of the newborn

The incidence of MetS at the follow-up study stratified with the birth weight of the offspring is illustrated in Fig. 1. The incidence of MetS was higher in women with GDM and an LGA (54.4%) than an AGA delivery (43.6%). Furthermore, the incidence of MetS in LGA study groups was three times higher in women with GDM as compared to the normoglycemic women. However, the incidence of MetS did not differ significantly in the non-GDM group between the AGA (24.5%) and LGA (18.8%) groups.

## Discussion

Our long-term study indicated that after an LGA delivery, the incidence of MetS is three times higher in women with GDM compared to those without GDM. Increased waist circumference was the only component of MetS in the non-GDM LGA group that was more prevalent than in the control group. Among the women with GDM, the limits of MetS in fasting glucose and HDL cholesterol were broken more often than in the control group. Overall, LGA delivery alone did not predict future MetS in women with normal glucose tolerance during pregnancy. In contrast, delivery of an LGA infant in women with GDM predicts future risk of MetS and thus risk for future cardiovascular disease.

Previously, only few studies have focused on the components of MetS separately after an LGA delivery without GDM. In our study, the mean waist circumference was significantly greater in women with GDM at follow-up in both birth weight categories. In addition, 81.3% of the women with a prior LGA delivery without GDM exceeded 80 cm waist circumference limit fulfilling the compulsory criterion of MetS. An explanation for this could be high pre-pregnancy BMI in this study group and genetic susceptibility to such body composition. These results are in agreement with a previous 18-years follow-up study showing that waist circumference and fasting glucose were the only significant components of MetS in mothers with LGA infants with or without GDM during their pregnancies [8]. In agreement with our results, no difference was observed in waist circumference in women with macrosomic (> 4 kg) or stillborn newborns as compared to age- and BMI-matched women without macrosomic deliveries in a 9-year follow-up study in women without previous GDM [19].

The development of dysglycemia and type 2 diabetes in women with a background of GDM supported by a large body of evidence [1, 2]. In accordance, a higher percentage of fasting glucose > 5.6 mmol/l was detected at follow-up in both GDM groups as compared to controls regardless of the birth weight category. However, no significant difference in fasting glucose was observed in women with a previous LGA delivery without GDM compared to controls. This is in agreement with a previous 2-year follow-up study, where fasting glucose levels did not differ between the non-GDM women with previous LGA and AGA deliveries [20]. Moreover, a 9-year follow-up study did not find any differences in fasting glucose concentrations in women with and without previous macrosomic newborns with absence of GDM during pregnancy [19].

In our study, LGA delivery without previous GDM did not predict later dyslipidemia as compared to the controls. Mean HDL cholesterol levels were lower and triglycerides slightly higher in women with GDM in both birth weight categories. However, concerning the lipid components of MetS, only low HDL cholesterol was more prevalent in the GDM groups than in controls. In agreement with our findings, a study performed 2 years after pregnancy revealed that no significant differences were observed between 18 women with LGA infants and 18 women with AGA infants with respect to lipids [20]. Correspondingly, a 9-year follow-up study demonstrated no differences in the incidence of dyslipidemia between 570 women with a history of macrosomia or stillbirth without GDM compared to age- and BMI-matched controls [19]. In contrast to these reports, a study of 48 women with previous birth of large infants and without glucosuria during pregnancy demonstrated that after 20–27 years postpartum these women had significantly lower concentration of HDL-cholesterol compared to age-, parity- and BMI-matched controls with birth weight < 4500 g [21]. Further, a study of 332 women with a prior LGA delivery reported lower HDL-cholesterol levels than in 2630 women with an appropriate-for-gestational-age (AGA) newborns in an age-adjusted model 18 years after pregnancy [8]. However, when adjusted for confounders the statistical significance was lost.

No studies have reported on the prevalence of the MetS high blood pressure criterion (≥130/≥85 mmHg) after an LGA delivery. We found no differences in this prevalence between the study groups. In agreement with our results, a prior macrosomic or LGA delivery in women without GDM did not predict later increased systolic or diastolic blood pressure in two-previous follow-up studies [19, 21].

Although some previous research has been carried out on components of MetS after an LGA delivery, the overall incidence of MetS has not been known. Our results show that 54.4% of women with GDM and an LGA delivery developed incident MetS during the follow-up, as compared to 43.6% in the AGA group. Interestingly, in women without GDM, the incidence of MetS was not higher with a previous LGA delivery as compared to the group with AGA delivery even though maternal BMI was higher in the LGA group. In this study, a considerable part of the women without GDM were overweight (BMI ≥ 25 kg/m^2^) in pre-pregnancy: 28.7% in the AGA and 51.1% in the LGA group (data not shown) as a result of risk-based screening for GDM. Therefore, it could be assumed that an LGA delivery in women without GDM is not predictive for later metabolic risk factors and MetS. Similarly, no association between an LGA delivery with the calculated 10-year CVD risk after adjustment for confounders was found in another study [8].

The strengths of the current study included the long-term follow-up of a well-characterized cohort of women, and the similar treatment received by all participants with GDM during pregnancy. It should be noted that in the present study, the GDM criteria in years 1989–2008 were tight especially regarding the fasting glucose value in OGTT. Thus, some women with GDM who would not be diagnosed with GDM using the current criteria were included as GDM women. This analysis has concentrated on women who were chosen from an obstetric population with risk factors for GDM potentially causing some selection bias. In addition, the study setting was cross-sectional at the time of follow-up OGTT, not longitudinal which would have been optimal to standardize the protocol. Notwithstanding its limitations, this study does suggest that even though all subjects have GDM risk factors, an LGA delivery does not predict later MetS in women without GDM.

In conclusion, the women without GDM were at a lower risk than those with GDM for MetS even with an LGA delivery. This probably reflects good maternal vascular health and its effects on birth weight. In contrast, women with GDM and a previous LGA delivery should be considered as a high-risk target group for prevention of future MetS and CVD.

## Conclusion

In summary, an LGA delivery without GDM was not significantly associated with future maternal MetS risk in the mean follow-up time of 7.3 years. High offspring birth weight in this group is likely to be related to maternal vascular health and genetic factors. In contrast, women with GDM who have had an LGA delivery should have a stringent follow-up after pregnancy to reduce the risk of future MetS and enhance women’s cardiovascular health.

## Abbreviations

AGA: Appropriate for gestational age
BMI: Body mass index
CVD: Cardiovascular diseases
GDM: Gestational diabetes
HDL: High density lipoprotein
IDF: International Diabetes Federation
LDL: Low density lipoprotein
LGA: Large for gestational age
MetS: Metabolic syndrome
OGTT: Oral glucose tolerance test
T1DM: Type 1 diabetes mellitus
T2DM: Type 2 diabetes
